# The Energetic Origins of Pi–Pi Contacts in Proteins

**DOI:** 10.1021/jacs.3c09198

**Published:** 2023-11-02

**Authors:** Kevin Carter-Fenk, Meili Liu, Leila Pujal, Matthias Loipersberger, Maria Tsanai, Robert M. Vernon, Julie D. Forman-Kay, Martin Head-Gordon, Farnaz Heidar-Zadeh, Teresa Head-Gordon

**Affiliations:** † Kenneth S. Pitzer Center for Theoretical Chemistry, University of California, Berkeley, California 94720, United States; ‡ Department of Chemistry, University of California, Berkeley, California 94720, United States; ▲ Department of Chemical and Biomolecular Engineering, University of California, Berkeley, California 94720, United States; ∥ Department of Bioengineering, University of California, Berkeley, California 94720, United States; ⊥ Department of Chemistry, Beijing Normal University, Beijing 100875, China; ¶ Department of Chemistry, Queen’s University, Kingston, Ontario K7L 3N6, Canada; □ Molecular Medicine Program, Hospital for Sick Children, Toronto, Ontario M5G 0A4, Canada; ▽ Department of Biochemistry, University of Toronto, Toronto, Ontario M5S 1A8, Canada; ○ Center for Molecular Modeling (CMM), Ghent University, 9052 Zwijnaarde, Belgium

## Abstract

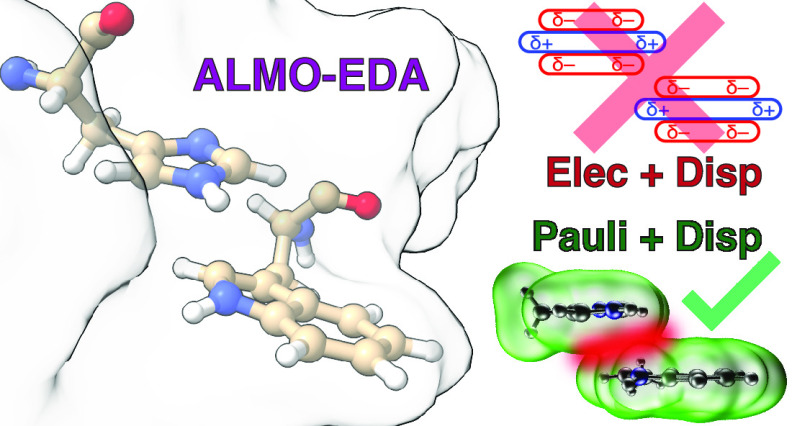

Accurate potential energy models of proteins must describe the many different types of noncovalent interactions that contribute to a protein’s stability and structure. Pi–pi contacts are ubiquitous structural motifs in all proteins, occurring between aromatic and nonaromatic residues and play a nontrivial role in protein folding and in the formation of biomolecular condensates. Guided by a geometric criterion for isolating pi–pi contacts from classical molecular dynamics simulations of proteins, we use quantum mechanical energy decomposition analysis to determine the molecular interactions that stabilize different pi–pi contact motifs. We find that neutral pi–pi interactions in proteins are dominated by Pauli repulsion and London dispersion rather than repulsive quadrupole electrostatics, which is central to the textbook Hunter–Sanders model. This results in a notable lack of variability in the interaction profiles of neutral pi–pi contacts even with extreme changes in the dielectric medium, explaining the prevalence of pi-stacked arrangements in and between proteins. We also find interactions involving pi-containing anions and cations to be extremely malleable, interacting like neutral pi–pi contacts in polar media and like typical ion–pi interactions in nonpolar environments. Like-charged pairs such as arginine–arginine contacts are particularly sensitive to the polarity of their immediate surroundings and exhibit canonical pi–pi stacking behavior only if the interaction is mediated by environmental effects, such as aqueous solvation.

## Introduction

Biopolymer chains from nucleic or amino acid building blocks arise from the creation of strong covalent bonds, whereas the three-dimensional structure of biomolecules is defined by the hierarchical organization of secondary and tertiary structural elements that are supported by the accumulation of many types of noncovalent interactions (NCIs).^1^ NCIs can be strongly stabilizing, e.g., salt bridges at physiological pH, highly directional in the case of hydrogen bonding, weak and isotropic in dispersion-dominated interactions between aliphatic groups, and highly cooperative through the influence of aqueous solvent, as observed in the hydrophobic effect.

Pi–pi contacts, typified by the electron-rich interactions between the delocalized pi-orbitals of aromatic groups, are ubiquitously observed in biological systems such as proteins,^2,3^ nucleic acids,^4^ and are a prevalent feature of molecular recognition of small molecule drugs that bind to active or allosteric sites.^5^ More recently, a computational analysis of the Protein Data Bank (PDB) observed that pi–pi contacts have biological implications for the ability of proteins to undergo phase separation, a phenomenon with significant importance in cellular organization and processes such as cell signaling and transcription.^6−11^ Pi–pi interactions at a more fundamental level combine the features of being energetically weak with respect to hydrogen bonding and yet are directional and cooperative, making them an especially interesting class of noncovalent interactions that need to be better understood given their biological relevance.

There are a number of ways to describe and identify pi contacts in proteins through their geometries to determine the preferred distance and orientation relevant to analyzing NCIs. McGaughey et al. examined a set of high-resolution X-ray crystal structures of nonhomologous proteins to determine the preferred positions and orientations between the aromatic side chains of the amino acids Phe, Tyr, His, and Trp.^3^ Furthermore, the relative orientations of the aromatic side chains were cataloged into different configuration types: off-centered parallel displaced (1p) and T-shaped (1t). Additionally, Pyrkov and co-workers^12^ investigated the role of stacking interactions in complexes of proteins with adenine and guanine fragments of ligands. Geometrical parameters such as displacement (d) and height (h) of one ring relative to the other and the angle γ calculated between the normal vectors of both rings were used to describe a stacking contact between two aromatic rings. More recently, Vernon and co-workers^8^ developed a geometric criteria to detect pi–pi contacts in their analysis of the PDB, which revealed that pi-stacking motifs in proteins have significant contributions from pi-orbital interactions between nonaromatic sp^2^-hybridized side-chains or the peptide bond itself. Although commonly associated with aromatic species, breaking the aromaticity in pi-networks has been associated with the seemingly paradoxical effect of enhancing pi-stacking interactions,^13^ showing that nonaromatic groups can play a structural role similar to the archetypal aromatic–aromatic interactions that epitomize the classic definition of pi–pi contacts.^8^

Geometric definitions such as the Vernon geometric criteria (VGC) naturally lead to a more fundamental question: what is a protein pi–pi contact from an energetic standpoint and what NCIs support its stabilization? In the gas-phase there is a competition between the electrostatic quadrupole moments of the interacting pi systems and the (London) dispersion interaction.^14^ The quadrupole moments of aromatic rings are generally repelled by one another, while dispersion is most favorable when the rings are cofacial, so a compromise emerges where the pi–pi contacts engage in offset stacking (parallel displaced) to retain most of the attractive dispersion forces while minimizing quadrupolar repulsions. This is the basis of the Hunter–Sanders model and has long been the principle paradigm for interpreting the structure of pi–pi contacts.^15−21^ The Hunter–Sanders model has recently been challenged on the basis that, apart from a tacit neglect of quantum electrostatics,^22−31^ it fails to describe simple pi–pi contacts like the benzene dimer.^32^ An alternative model based on a competition between Pauli repulsion (the repulsive interaction between electron clouds caused by the antisymmetry requirement of the wave function) and dispersion has proven to be far more successful in describing the various geometries of pi–pi contacts.^32,33^ The van der Waals model suggests that the parallel-displaced arrangement of pi–pi contacts can be described without invoking electrostatics and seems to be consistent with recent theoretical and experimental work from microwave spectroscopy on polycyclic aromatic hydrocarbons to the serrated stacking pattern observed in covalent organic frameworks.^34,35^

In this work, we quantify the energetic origins of pi–pi contacts in proteins using energy decomposition analysis (EDA).^36,37^ An EDA separates intermolecular interactions into separate contributions that can be associated with different physical driving forces such as permanent and induced electrostatics, Pauli repulsions, dispersion, and dative interactions, whose relative magnitudes provide an objective fingerprint that characterizes the interaction. Thus, EDA can be used to test the underlying assumptions of the Hunter–Sanders and van der Waals models for recognizing a pi–pi contact and understanding its energetic origins. In particular, we use absolutely localized-molecular-orbital EDA (ALMO-EDA)^37,38^ within a density functional theory (DFT) framework to understand the driving forces behind NCIs in pi–pi contacts between tyrosine (Tyr), phenylalanine (Phe), tryptophan (Trp), histidine (His), glutamine (Gln), asparagine (Asn), glutamic (Glu), aspartic (Asp), and arginine (Arg) amino acids, and including the backbone peptide moiety. Furthermore, ALMO-EDA has been extended to incorporate effects of a solution-phase environment through use of continuum models, yielding insights into how environmental effects modulate interactions.^39^ Hence, we perform ALMO-EDA calculations in both the gas phase and in the presence of solvent dielectric via a simple polarizable continuum model (PCM) that accounts for electrostatic screening effects due to the environment^40^ in order to understand environmental effects on pi–pi contact stabilization.

The EDA is applied to structural protein motifs derived from polarizable force field simulations as they can provide a better physical model than fixed-charge force fields for capturing both folded proteins and proteins with intrinsic disorder.^41^ In particular, many-body potentials can simultaneously describe solution experiments for the folded states of 7 globular proteins, the TSR4 domain that has regions of disorder, the fully disordered Hst 5 peptide, as well as the disorder to order transition as temperature is lowered for the (AAQAA)_3_ peptide.^41^ In this study, we utilize the AMOEBA polarizable force field^41^ simulations to generate a benchmark suite of putative pi–pi contacts that are extracted using VGC^8^ from the TSR4 domain (1vex),^42^ the sugar-binding protein DC-SIGN (2xr6),^43^ and a serine protease (1arb).^44^ The resulting pi–pi contacts configurations are then analyzed using EDA to dissect the interaction energy into physically intuitive contributions including permanent electrostatics, polarization, charge transfer, Pauli repulsion, and dispersion,^37^ lending insight into the physical origins of pi-contact motifs.

Overall, we find that Hunter–Sanders pi–pi contacts appear to be less common in proteins (occurring only 3% of the time in our data) than van-der-Waals-type pi–pi contacts. Moreover, Hunter–Sanders-type pi–pi contacts contribute less to the overall stability of the protein due to their repulsive electrostatics and overall weaker interactions due to larger distances between fragments. This is a significant finding of this work, as the interactions of pi–pi contacts in proteins have heretofore been discussed under the tacit assumption that the Hunter–Sanders-type pi–pi contact is the most prevalent,^3,14,45^ but our results imply that it is instead in the minority. The astounding abundance of van-der-Waals-type pi–pi contacts in proteins suggests that a shift away from the Hunter–Sanders paradigm of pi-stacking in proteins could greatly benefit force field design principles, phase separation in biocondensates, and the qualitative understanding of pi–pi interactions more broadly.

## Methods

### Geometrical Definitions of Pi–Pi Contacts

The VGC procedure^8^ identifies pi–pi contacts based on the following protocol: (1) Identify sp^2^-planes and record coordinates of the peptide backbone amide group, i.e., the −HN–C=O fragment, as well as side-chain fragments of 9 amino acids including Arg, His, Asp, Glu, Asn, Gln, Phe, Tyr, and Trp. (2) Measure the distance between the sp^2^-planes. This is done by first projecting the planar surfaces (defined by the constituent atoms) to a distance of 1.7 Å (the van der Waals radius of carbon) along each plane’s normal vector and then measuring the pairwise distance of (projected) atoms of two planes. If at least two pairs of atoms (each from different planes) have a distance ≤1.5 Å, go to the next step, otherwise, there are no pi–pi contacts between these planes. (3) Measure the angle between the sp^2^-planes. A pi–pi contact is identified between two planes if the dot product of their normal vectors is ≥0.8 (i.e., the angle between the planes ranges from 0° to ∼36°). Due to the nature of the hard cutoffs employed in VGC, we anticipate appreciable sensitivity to the selection of model parameters. Note that VGC is entirely geometrical; therefore, there is no consideration of residue charge or environment. Also, it is pertinent to understand that this geometrical definition was developed to identify the sequence location of planar surface area contacts for structures found in the PDB, and the cutoffs were based on statistical concerns related to handling lower-resolution X-ray crystal structures and not for energetic reasons.

### Construction of the Protein-Fragment Database

**Figure 1:**
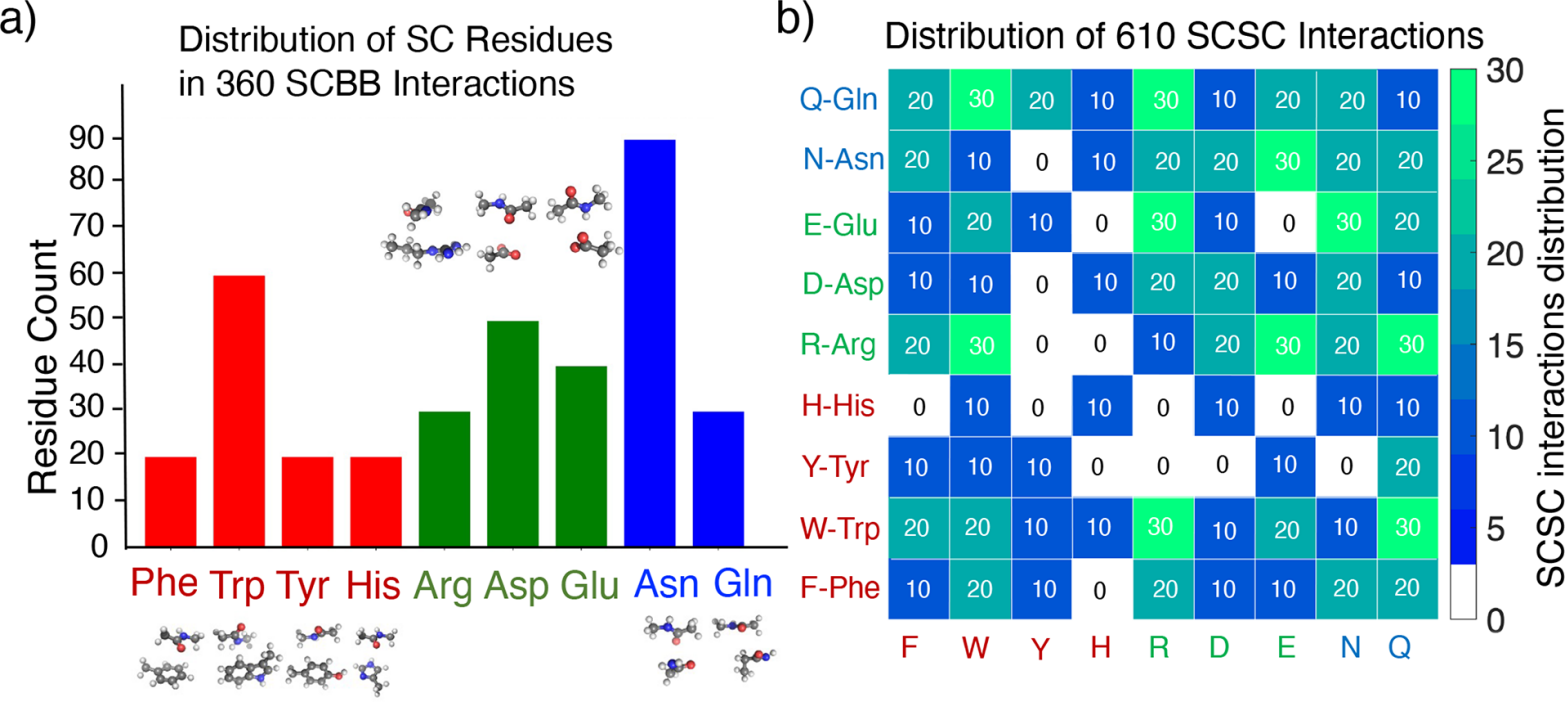
Distribution of side-chain (SC) residues in our protein fragment database. Side-chain–backbone (SCBB) interactions (left) and side-chain–side-chain (SCSC) interactions (right). The backbone peptide group is terminated with methyls, and side chains are terminated at C_β_ with hydrogen atoms.

From a series of whole frame snapshots of one μs molecular dynamics trajectories^41^ of the TSR4 domain (1vex),^42^ the sugar-binding protein DC-SIGN (2xr6),^43^ and a serine protease (1arb),^44^ we used the VGC to capture all relevant noncovalent pi–pi interactions between side-chain (SC) and backbone (BB) fragments. Specifically, we focused and extracted snapshots to guarantee a well-balanced database of fragments with and without pi–pi contacts according to the VGC. Our pi-contact database includes 200 backbone–backbone (BBBB), 360 side-chain–backbone (SCBB), and 610 side-chain–side-chain (SCSC) interactions, of which 94, 189, and 256 of these were identified as pi–pi contacts, respectively. Methylacetamide is used as the host for pi–pi interactions in the BB subset, whereas Arg, His, Asp, Glu, Asn, Gln, Phe, Tyr, and Trp amino-acid side chains are represented in the SC subset. We group these amino acids into 3 classes: aromatic (Phe, Trp, Tyr, and His), hydrophilic (Asn and Gln), and charged (Arg, Asp, and Glu). Figure 1 shows the distribution of SC residues in our database.

### Energy Decomposition Analysis (EDA)

Among existing quantum mechanical methods for decomposing the intermolecular interaction energy into physically motivated components,^37,46,47^ we used the energy decomposition analysis (EDA) based on absolutely localized molecular-orbitals (ALMO) scheme.^37,38,48^ The ALMO-EDA decomposes the total interaction energy, *ΔE*_int_ into five terms:
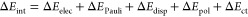
1where

- *ΔE*_elec_ (Electrostatics) describes permanent electrostatics via a classical Coulombic interaction of the total charge distributions of the interacting fragments (nuclei and electrons).
- *ΔE*_Pauli_ (Pauli repulsion) arises from the Pauli exclusion principle of electrons and captures the energetic penalty of abiding by wave function antisymmetry between fragments.
- *ΔE*_disp_ (Dispersion) is an attractive interaction due to correlated fluctuations of electrons.
- *ΔE*_pol_ (Polarization) describes the distortion of the electron density in the electrostatic potential of other molecules.
- *ΔE*_ct_ (Charge transfer) is the energy lowering associated with orbital mixing across different fragments and is often referred to as donor/acceptor interactions.

Throughout this work, electrostatics and Pauli repulsion terms will often be considered together as the “frozen” energy, and the polarization and charge-transfer terms will be grouped as “orbital” interactions.
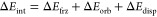
2See ref (37) for a detailed discussion of each component and for a detailed review of the ALMO-EDA method.

We employ the recently developed ALMO-EDA(solv) scheme in order to incorporate the effects from a dielectric environment throughout each step of the EDA procedure.^39^ The ALMO-EDA(solv) scheme allows for a direct evaluation of solvent effects supplied by self-consistent reaction field models such as PCM “on-the-fly” as opposed to a posteriori corrections that are typically used. This amounts to solvent corrections entering each term in eq 1 by,
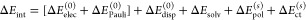
3where all quantities with a superscript (*s*) are computed in consideration of implicit solvent, and those with a superscript (0) are the corresponding gas-phase values. The solvation correction to *ΔE*_frz_^(0)^ (terms in square brackets), is denoted separately and is given by,
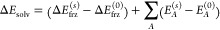
4where *E*_*A*_ is the energy of isolated monomer *A* and,
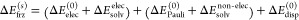
5where *ΔE*_solv_^elec^ and *ΔE*_solv_^non-elec^ are the electrostatic and nonelectrostatic components of the solvation energy. Notably, it is assumed that *ΔE*_disp_^(*s*)^ ≈ *ΔE*_disp_^(0)^, e.g., the presence of implicit solvent does not impact the dispersion term. Finally, the polarization and charge-transfer terms are computed relative to Δ*E*_frz_^(*s*)^ and Δ*E*_full_^(*s*)^, respectively (where Δ*E*_full_^(*s*)^ is the total energy of the complex in the presence of implicit solvent). This leads to an overall interaction energy that incorporates implicit solvent,
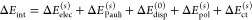
6

We employ a simple PCM model that incorporates only the influence of electrostatics, so our solvated frozen energy in eq 5 incorporates only electrostatic screening effects. We found that including nonelectrostatic terms does not influence the qualitative interpretation of the results presented herein, and we have made these data available in the Supporting Information.

### Computational Protocol

All ALMO-EDA calculations were performed with Q-Chem software package (version 5.2)^49^ at the ωB97X-V/def2-TZVPD level of theory. The ωB97X-V functional^50^ is a range-separated hybrid Generalized Gradient Approximation (hybrid-GGA) with VV10^51^ nonlocal correlation and is consistent with best practices for intermolecular interactions.^52−55^ The exchange-correlation potential was evaluated on a fine Lebedev quadrature using 99 radial and 590 angular grid points (99, 590) while a smaller (50, 195) grid was used for evaluating the VV10 functional.

The IOData^56^ package was then used to parse and analyze the ALMO-EDA results. The Procrustes^57^ library was used for the alignment of some dimer fragments prior to performing EDA calculations. Solvent effects were included implicitly via a conductor-like PCM formalism, using a dielectric constant of ε = 78.39 for water (henceforth referred to as “PCM water”) and a cavity constructed using a solvent accessible surface with a probe radius of 1.4 Å to prevent the PCM charges from interspersing between close-contact moieties.^40^ In the Supporting Information, we include additional ALMO-EDA data with ε = 2 to emulate the hydrophobic pockets of a generic protein.

## Results

**Figure 2:**
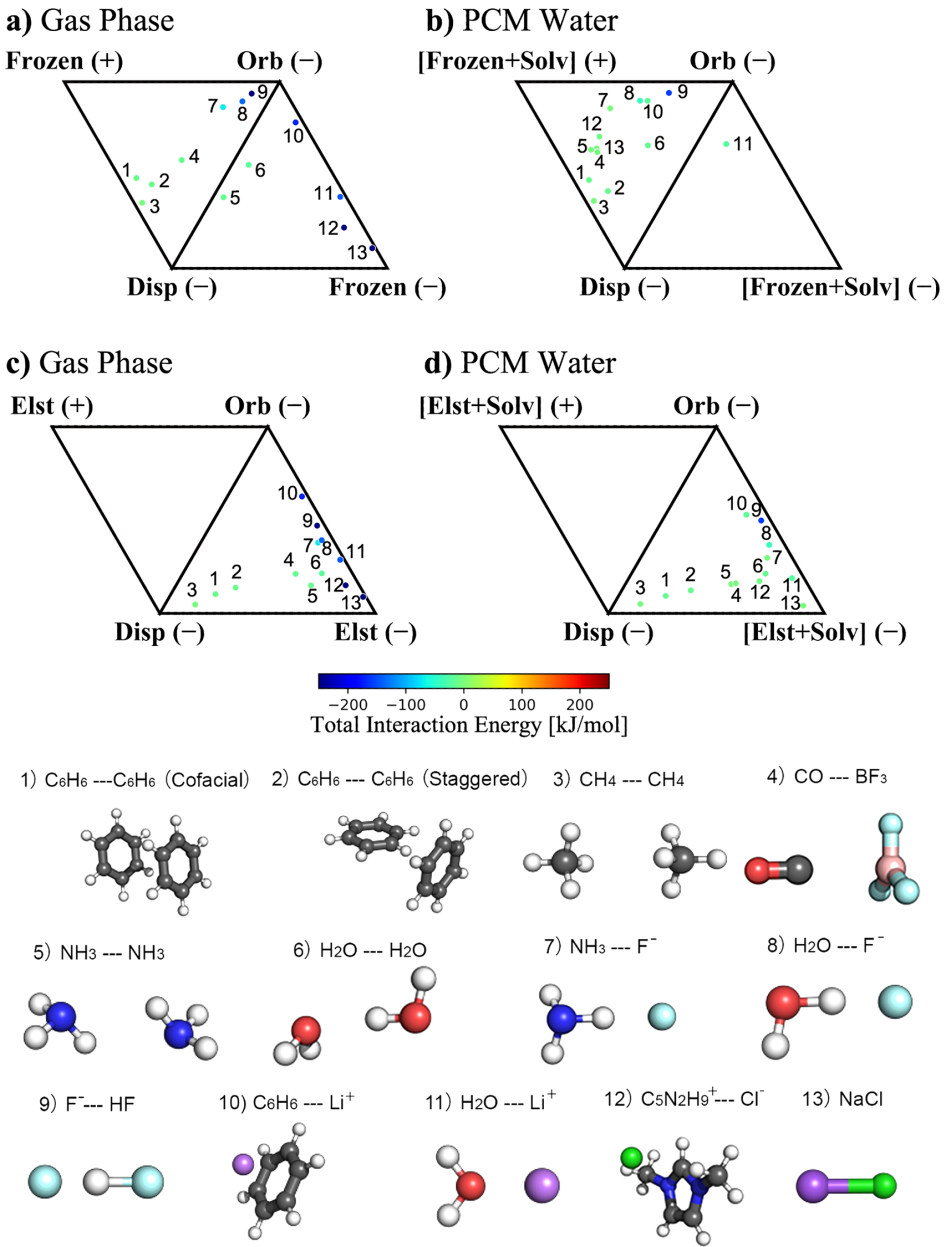
Ternary diagrams generated from ALMO-EDA of various chemical interactions for common molecules. Ternary diagrams utilizing the Frozen (Frz) interaction (a) in the gas phase and (b) with the inclusion of a high dielectric environment from PCM. The (c) and (d) plots subtract out the Pauli repulsion from (a) and (b), leaving just permanent electrostatics (Elst), respectively. The corresponding plots with a dielectric = 2.0 environment from PCM are included in Figure S2. These provide a reference throughout our discussion of pi–pi interactions herein.

Interaction energy components from ALMO-EDA are analyzed using ternary diagrams.^77^ The position of each point on the ternary diagram represents the ALMO-EDA ratio,
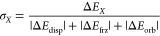
7where *X* = disp, orb, or frz. Note that the interaction energy component in the numerator retains its sign; therefore, while Δ*E*_disp_ and Δ*E*_orb_ are always stabilizing, we must dedicate two vertices to Δ*E*_frz_ to distinguish stable and unstable states. Figure 2 shows the gas phase ternary diagram for common examples such as the benzene and methane dimer that are dispersion-dominant, ionic interactions such as found for NaCl that are electrostatic-dominant, and hydrogen bonding interactions (e.g., water dimer) that are mixed dispersion-orbital interactions.

In addition, the ternary plots will provide analysis for both the frozen energy (Δ*E*_frz_) illustrated in Figure 2a and 2b and just the electrostatics without the Pauli repulsion contribution (Δ*E*_elst_) portrayed in Figure 2c and 2d to gain insight into how the systems behave under both circumstances. Significantly, Figure 2a shows that the canonical aromatic–aromatic (points 1 and 2) or aliphatic interactions (point 3) have expected favorable dispersion, and the frozen energy is unfavorable. However, Figure 2c reveals that the underlying electrostatic interactions in neutral, aromatic pi–pi interactions are in fact attractive (points 1–3 lie in the Elst(−) domain), which is strictly antithetical to the Hunter–Sanders model which proposes that the dominant contribution from electrostatics is repulsive. Notably, favorable electrostatics and a repulsive frozen energy for the overall aromatic pi–pi interaction adds to a growing amount of evidence that the Hunter–Sanders (HS) model can fail,^22,26,33,58^ and supports an alternative model based on the van der Waals interactions that was proposed by Carter-Fenk and Herbert (CFH).^33^ Finally, we consider how frozen/electrostatics shift in the presence of a high dielectric solvent (Figure 2b and 2d) for this classic set of molecules which can shift the classification yet again. Thus, ternary diagrams that are generated as we analyze pi–pi contacts in proteins can be referred back to Figure 2 to provide a touchstone for common NCIs and their environments.

### Classifying Pi–Pi Contacts in Diverse SCSC Interactions

**Figure 3:**
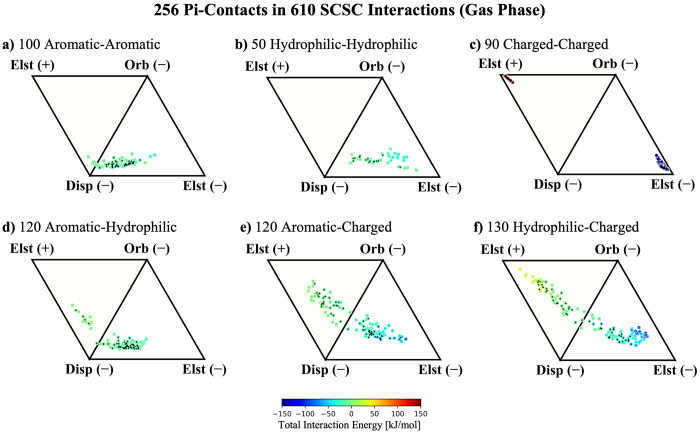
ALMO-EDA energy components for 610 SCSC interactions in the gas phase broken into dispersion, orbital, and electrostatic interactions. a) 100 aromatic–aromatic with 46 pi–pi contacts, b) 50 hydrophilic–hydrophilic with 16 pi–pi contacts, c) 90 charged–charged with 26 pi–pi contacts, d) 120 aromatic–hydrophilic with 54 pi–pi contacts, e) 120 aromatic–charged with 60 pi–pi contacts, and f) 130 charged–hydrophilic with 54 pi–pi contacts. The total interaction energy ranges from a) −23 to −2, b) −41 to 5, c) −463 to 298, d) −24 to 13, e) −75 to 5, and f) −94 and 32 (in kJ/mol). The 256 interactions with pi–pi contacts are marked with a black diamond.

We begin our analysis of pi–pi contacts for gas-phase interactions that are unadulterated by environmental effects of a continuum model of solvent. Initially, we consider the 610 side-chain–side-chain (SCSC) fragments, as they feature the most diverse range of NCIs for pi–pi interaction groups, including many motifs that share strong similarities to archetypal pi–pi contacts that have been studied thoroughly in gas-phase quantum chemistry. Specifically, this subset features aromatic–aromatic, hydrophilic–hydrophilic, charged–charged, aromatic–hydrophilic, aromatic–charged, and hydrophilic–charged NCIs, allowing us to partition the ALMO-EDA results in Figure 3 to compare and contrast the interaction profiles of these various SCSC motifs, that will later inform side-chain–backbone (SCBB) and backbone–backbone (BBBB) pi–pi interactions analyzed below.

Figure 3a considers the SCSC aromatic–aromatic interactions, as these can be straightforwardly compared with the canonical pi–pi interactions of the gas-phase benzene dimer, by first considering only the electrostatics of the frozen term (i.e., ignoring Pauli repulsion). Neutral aromatic–aromatic pi–pi contacts show a very consistent clustering toward dominant dispersion interactions and favorable electrostatics. This is consistent with previous studies of Phe–Phe interactions^45^ and the benzene–benzene case described above, but the larger scope of our study establishes attractive electrostatics as a general phenomenon in biological pi–pi systems that extends beyond Phe residues.

Moreover, this finding is particularly significant in light of the competing pictures of the HS and CFH models of pi-stacking. Favorable electrostatics between aromatic motifs in Phe, Tyr, Trp, and His implies a deviation from the classical quadrupole repulsion that can be understood as a charge penetration effect.^22,26,33^ Charge penetration is defined as the interspersion of electron clouds when molecules interact at short range, causing electrons on each molecule to experience an attractive interaction with the nuclei of the other (a descreening of electron/nuclear attraction). We note that charge penetration is of considerable biological importance in the stacking interactions between DNA base pairs and has been implicated in the large errors of molecular mechanics potentials for short-range pi–pi interactions.^59−61^ In particular, charge penetration can also be viewed as a natural consequence of the van der Waals model of pi–pi interactions, because the van der Waals picture is valid in the close-contact limit where Pauli repulsion and dispersion tend to dominate. In this limit, the large surface area of the pi-system conspires with the short-range nature of the interaction to amplify electrostatic attraction through the charge penetration effect, often in spite of repulsive quadrupole moments.^33,58^

Moving on to the SCSC hydrophilic–hydrophilic pi–pi contact interactions (Figure 3b), we find that they take on the same qualitative trends as those of aromatic–aromatic ones, making them more or less indistinguishable from the nominal case of pi-stacking. Their interaction profile is somewhat shifted toward favorable electrostatics and away from dispersion, but this is likely a simple consequence of geometry as nonaromatic motifs generally engage in more favorable charge penetration at larger distances.^13^ The aromatic–hydrophilic interactions in Figure 3d obey a similar trend, being largely dominated by attractive electrostatic and dispersion interactions. Here, the geometric model identifies four instances of pi–pi contacts out of 120 that have a positive contribution from electrostatics. These might be considered HS-type pi–pi interactions because the multipolar electrostatics are the most significant and their opposing multipole moments lead to net destabilization.

We note that the specific parameters used in the VGC definition may influence the resultant ratios of CFH- and HS-type pi–pi contacts. However, by considering all of the points in Figure 3, it is clear that even if the distance parameter is taken to the limit of large *R*, where all systems that at least meet the angular criteria are counted as pi–pi contacts, the number of possible HS-type pi–pi contacts has a ceiling that is still much lower than the number of van der Waals contacts. Therefore, we anticipate that the conclusion that HS-type pi–pi contacts are in the minority holds irrespective of the particular application of distance thresholds in the geometric model.

Next, we consider the SCSC systems that feature charged residues in Figure 3c, 3e, and 3f. The interaction profile in Figure 3c reveals that the energy contributions for 90 charged–charged motifs are what might be expected from a system of two interacting ions (Figure 2). No significant contribution in the charged–charged interactions comes from anything but the classical electrostatics, and their interactions are all very strong, with the most stable being roughly −110 kcal/mol, rivaling the strength of a chemical bond. Pi–pi interactions tend to be much weaker than this, and with nothing to distinguish the charged–charged interactions from those of simple ions, there is little sense in discussing them as pi–pi contacts as it obfuscates the true nature of the interaction. Later on, we will contrast this with polar environments that might be supplied by acid or base residues or solvent exposure.

An interesting middle ground between typical pi–pi and ion–ion interactions is found in the SCSC aromatic–charged and hydrophilic–charged interactions in Figure 3e and 3f. Although not nearly as potent as those in charged–charged motifs, these aromatic/hydrophilic–charged interactions exhibit far more orbital and electrostatic effects than typical pi–pi contacts. There is a balance between orbital and dispersion interactions in these systems, which is sensible as the lobes of pi–electron density are highly polarizable, so in the presence of a permanent charge, *ΔE*_orb_ should be expected to be more dominant. Overall, the aromatic/hydrophilic–charged motifs have interaction profiles that strongly resemble cation/anion–pi (or ion–pi) interactions.^62,63^ The ion–pi interactions between aromatic/hydrophilic–charged pi systems in proteins are similar to the Li^+^···benzene interaction in Figure 2, but because Arg, Asp, Glu, Asn, and Gln are substantially larger than Li^+^, there is a steric effect that keeps them significantly further away from the other monomer. This steric effect reduces the magnitude of polarization and charge transfer, but the larger size of the interacting monomers also has a stabilizing influence in the form of a larger dispersion interaction, as dispersion is an extensive quantity of a system. These two effects conspire to form a balance between dispersion and orbital interactions in ion–pi motifs in nonpolar places inside proteins.

**Figure 4:**
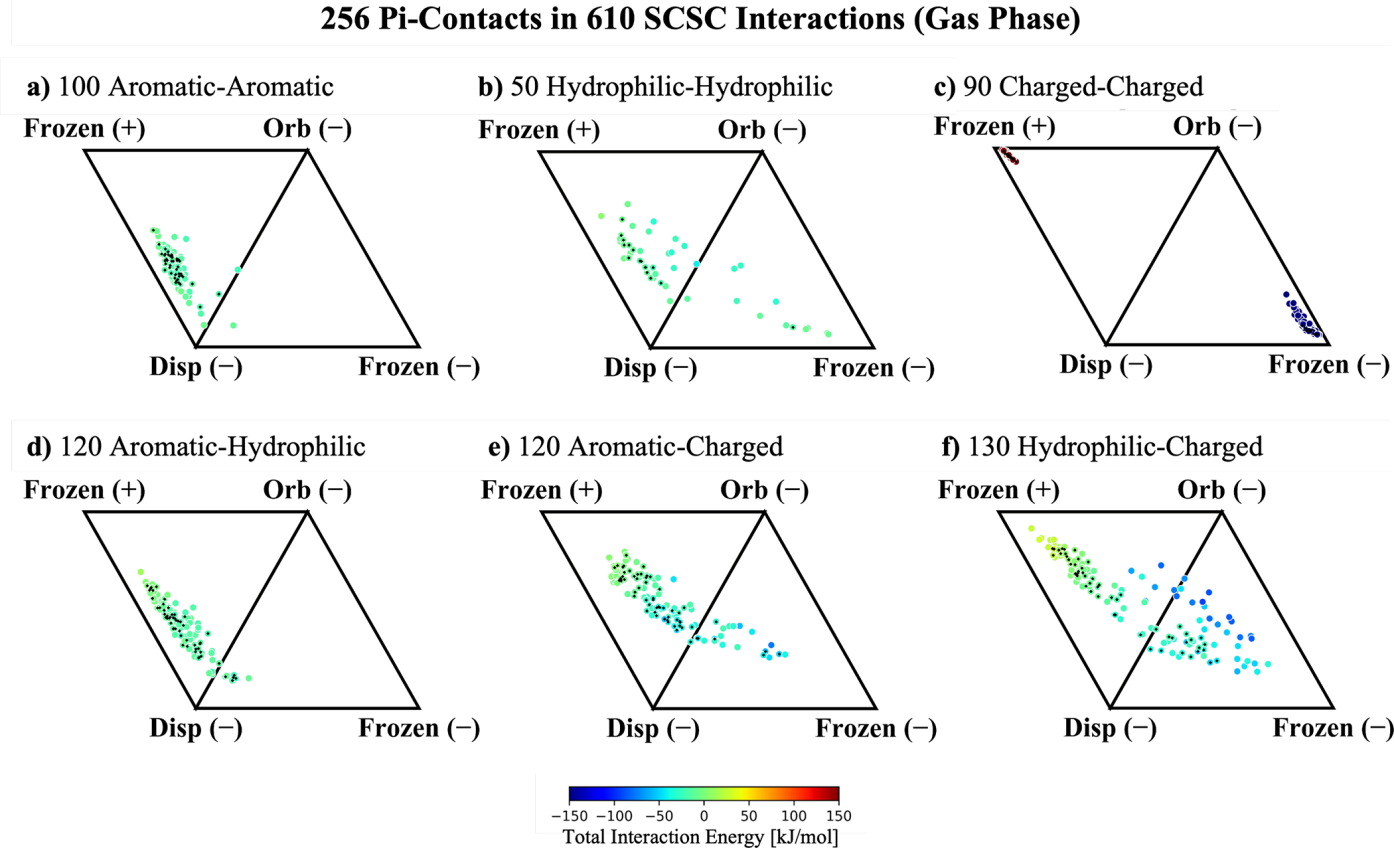
ALMO-EDA energy components for 610 SCSC interactions in the gas phase broken into dispersion, orbital, and frozen (electrostatic and Pauli repulsion) interactions. a) 100 aromatic–aromatic with 46 pi–pi contacts, b) 50 hydrophilic–hydrophilic with 16 pi–pi contacts, c) 90 charged–charged with 26 pi–pi contacts, d) 120 aromatic–hydrophilic with 54 pi–pi contacts, e) 120 aromatic–charged with 60 pi–pi contacts, and f) 130 charged–hydrophilic with 54 pi–pi contacts. Vertices labeled Frozen(+) and Frozen(−) contain positive and negative contributions from Δ*E*_Frz_ = Δ*E*_Elst_ + Δ*E*_Pauli_. The 256 interactions with pi–pi contacts are marked with a black diamond.

Now that the interaction profile of each type of pi–pi contact under consideration has been evaluated within the scope of electrostatics, dispersion, and orbital interactions, we next approach the full reconstruction of the interaction energy by adding Pauli repulsion back into the frozen term. The results in Figure 4 reveal that Pauli repulsion is substantially more important for all of the neutral SCSC subsets of pi–pi contacts (aromatic–aromatic, aromatic–hydrophobic, and hydrophobic–hydrophobic). The dominance of Pauli repulsion is made clear by the fact that the attractive electrostatic contribution gives way to an overall repulsive frozen energy, implying that |Δ*E*_Pauli_| > |Δ*E*_Elst_|. It is also interesting to note that in the case of hydrophilic–hydrophilic subsystems, the orbital interactions become more significant once Pauli repulsion is taken into account, increasing the scope of the hydrophilic–hydrophilic interaction profile. Overall, the shift toward repulsive frozen energies clarifies that dispersion and Pauli repulsion (the van der Waals interactions) are indeed the most prominent in neutral pi–pi contacts.

The results for charged systems in Figure 4c, 4e, and 4f make yet another case for considering ion–pi and ion–ion interactions as distinct from pi-stacking. In each case, the inclusion of Pauli repulsion leaves the ternary diagram qualitatively unperturbed, indicating that the van der Waals interactions are much less significant for these systems. One noticeable change is in the distribution of the systems that were identified as pi–pi contacts within the ion–pi subsets (Figure 4e and 4f), which do shift toward positive frozen energies when Pauli repulsion is included. This shift is not accompanied by similar changes in the overall interaction profile (the ion–pi interactions still strike a balance between dispersion, orbital interactions, and electrostatics), suggesting that Pauli repulsion remains important but does not necessarily outcompete the electrostatic contributions, as was evident in the neutral systems. This shift in the pi–pi contact distribution is likely due to the small distance between pi systems because Pauli repulsion increases exponentially with the overlap of charge densities. Despite these changes, the overall interaction profiles of ion–ion and ion–pi systems are still primarily electrostatics/orbital driven; thus, it remains sensible to discuss ion–ion and ion–pi interactions on a different footing than those of neutral pi–pi contacts in nonpolar environments.

### Impacts of the Protein vs Solvent Environment

The environment in proteins is complex and variable, so we have modeled the effects of embedding each pi system into a uniform dielectric potential with varying dielectric constants. The gas-phase data presented above are one extreme where ε = 1 (representative of hydrophobic regions in proteins), while the other extreme in this work is the dielectric of pure water (ε = 78.39). We also report the effects of a dielectric that is more consistent with those usually found within a heterogeneous protein (ε = 2) in the Supporting Information. While these results show the general trends that are to be expected from embedding these pi systems into a more complex environment, we acknowledge that the results should only be interpreted qualitatively due to the significant approximations that are made when employing polarizable continuum models.

**Figure 5:**
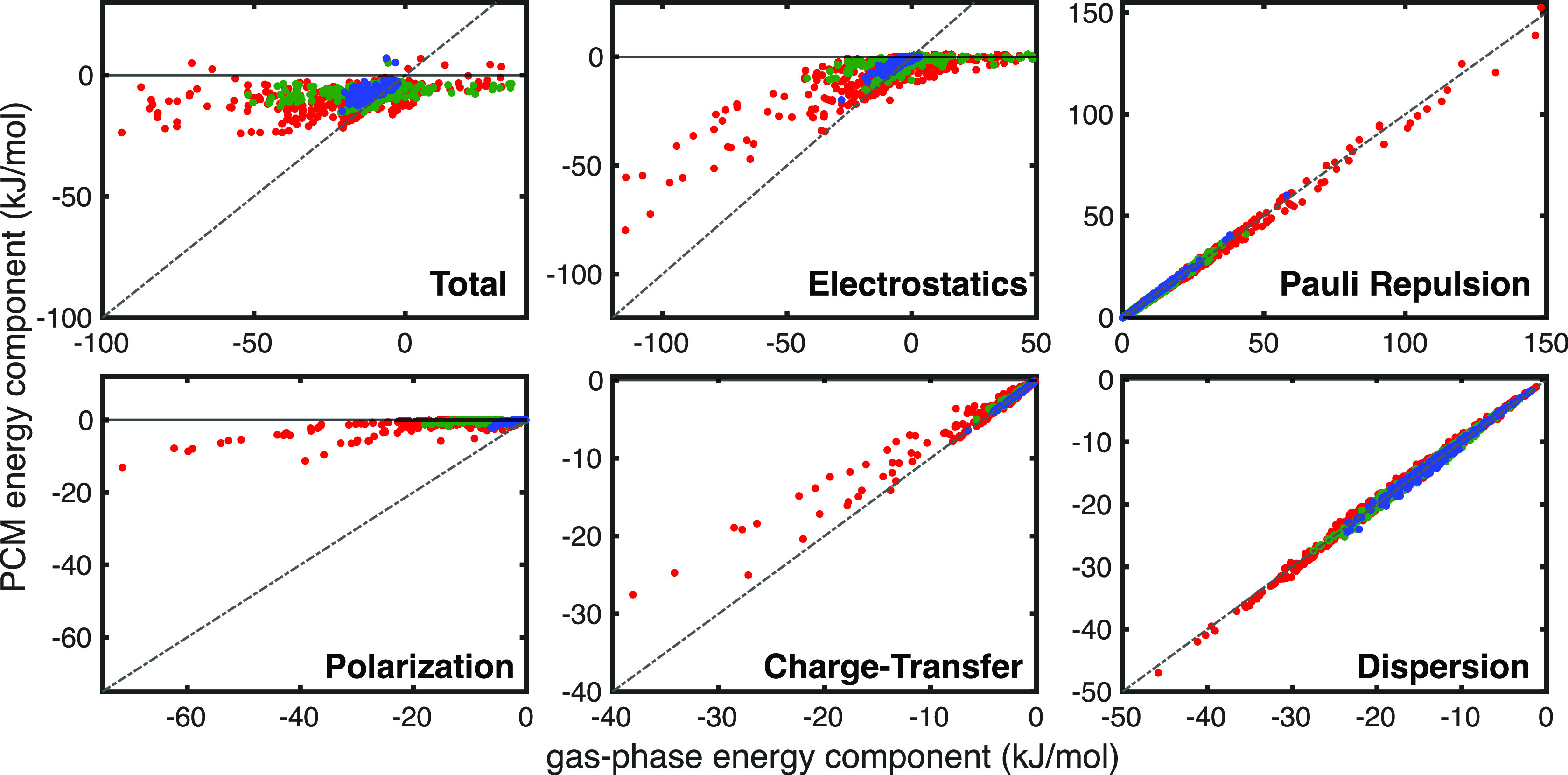
Parity plots for ALMO-EDA components in the gas phase (*x*-axis) and PCM with dielectric ε = 78.39 (*y*-axis). The data are color-coded according to (red) SCSC, (green) SCBB, and (blue) BBBB subsets. Parity is shown as a gray dashed line. The solvation correction, *ΔE*_solv_, is included within the ALMO-EDA(solv) electrostatic term.

To understand the impact of PCM on the ALMO-EDA results, we construct parity plots for each interaction energy component along with the total interaction energy. The results in Figure 5 show significant changes in the total interaction energy due to PCM. This difference quite evidently manifests due to electrostatic screening effects, which diminish the contributions of permanent electrostatics and polarization to the total interaction energy, while the Pauli repulsion, dispersion, and, to a lesser extent, charge-transfer interactions are largely unaffected by PCM. This can be quantitatively validated by comparison of the interaction energy difference, Δ*E*_int_(PCM) – Δ*E*_int_(gas), with the change due to electrostatic screening in Figure 5, which reveals that effectively all of the difference in interaction energy due to PCM comes from the screening of electrostatics and polarization components. The marginal changes in Pauli repulsion and dispersion should be expected as these interactions are sensitive only to the subtle changes in electron density polarization that come from the solvent. These small changes in the van der Waals interactions are accompanied by large changes in electrostatic effects, which implies that HS-type pi–pi contacts should be drastically affected by the change in the electrostatic environment when going from a hydrophobic pocket to a solvent-exposed region in the protein, while CFH-type pi–pi contacts should be left intact.

**Figure 6:**
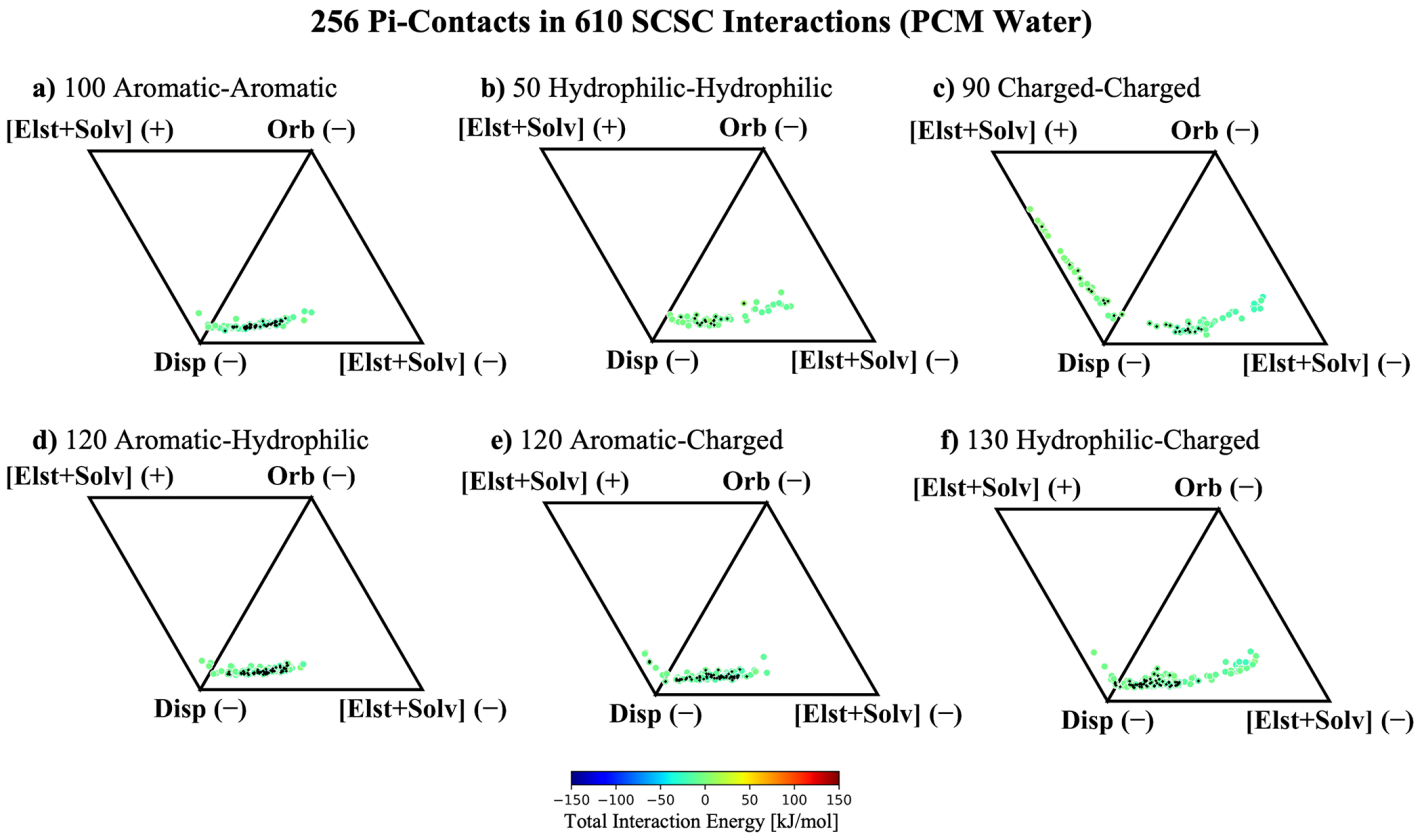
ALMO-EDA energy components for 610 SCSC interactions in a PCM environment broken into dispersion, orbital, and electrostatic interactions. The PCM water counterpart of Figure 4. Similar plots with a dielectric constant of 2.0 are provided in Figure S3. The 256 interactions with pi–pi contacts are marked with a black diamond.

As the presence of electrostatic screening clearly changes the interaction profile, we now revisit the ternary diagrams to study the impact of the environment on the distribution of interaction energy components, first considering the exclusion of Pauli repulsion in Figure 6. Notably, the aromatic–aromatic SCSC interactions in Figure 6a are basically unperturbed by the presence of the large dielectric field of water, still exhibiting clustering around attractive dispersion and electrostatics. The unwavering nature of the aromatic–aromatic interaction profile despite large changes in dielectric is consistent with the CFH model, as Pauli repulsion and dispersion do not change significantly in response to the electrostatic environment.^32^ The hydrophilic–hydrophilic and aromatic–hydrophilic interactions in Figure 6b and 6d also remain relatively unchanged with the introduction of solvent dielectric. This is a crucial finding, as it implies that neutral pi–pi contact interactions should be relatively unperturbed by the dielectric medium, leading to consistent contact geometries regardless of the polarity of the immediate protein or solvent environment.

Once again, the charged moieties in Figure 6c, 6e, and 6f exhibit unique behavior that should be discussed separately from that of the neutral systems. Interestingly, the aromatic–charged and hydrophilic–charged systems that were identified as ion–pi interactions in the gas phase exhibit an interaction profile that collectively looks much more akin to typical aromatic–aromatic/aromatic–hydrophilic interactions within PCM. This is easily explained, as structurally these species all qualify as pi–pi contacts within VGC, and Figure 5 shows that the polarization interactions are quenched within a high-dielectric medium. After the orbital interactions are effectively nullified, the remainder of the ion–pi interactions essentially look like neutral–neutral pi-stacking. This has the important implication that ion–pi interactions in solvent-exposed domains on the exterior of a protein may take on the role of more typical van-der-Waals-type pi–pi contacts. These results, which imply that ion–pi interactions can be tuned from the limit of neutral pi–pi contacts in polar environments to the opposite limit of ion–pi interactions in nonpolar solvent, are also consistent with examples of electrostatic tunability of ion–pi interactions reported elsewhere in the literature.^64−67^

On the other hand, the ion–ion interactions in Figure 6c remain quite influenced by the sign of their electrostatic monopole moments. However, with the quenching of polarization effects, these species can be said to be dominated by electrostatics and dispersion. While electrostatic screening of solvent brings ion–ion interactions into closer alignment with typical pi–pi contacts, it remains clear that the sign of the monopoly moments considerably impacts the overall interaction. This suggests that ion–ion interactions are somewhat less tunable than ion–pi contacts because where ion–pi interactions can be tuned between hydrophobic and highly electrostatically driven limits, the ion–ion interactions retain their strong dependence on their innate electrostatic charge regardless of their surroundings.

**Figure 7:**
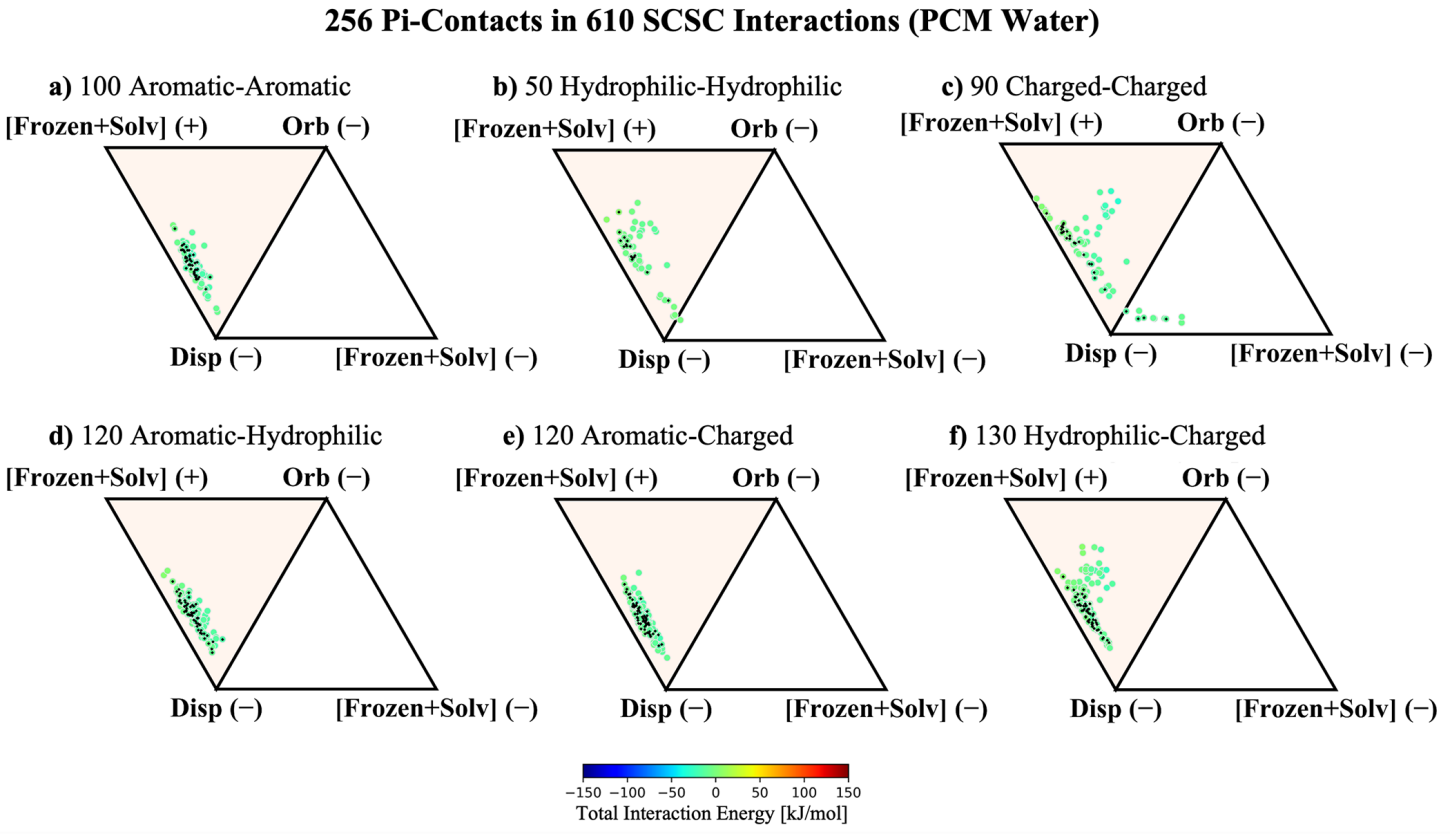
ALMO-EDA energy components for SCSC interactions in a PCM environment broken into dispersion, orbital, and frozen interactions. PCM water counterpart of Figure 4. Similar plots with a dielectric constant of 2.0 are provided in Figure S4. The 256 interactions with pi–pi contacts are marked with a black diamond.

Adding the Pauli repulsion term back into the signed vertices in Figure 7 reveals that all of the systems that engage in pi-stacking interactions in solution (neutral pi–pi and ion–pi systems) behave like van der Waals pi–pi contacts. Their interaction profiles are dominated by dispersion and Pauli repulsion, the latter of which completely outcompetes the attractive electrostatic interactions. The ion–ion systems in Figure 7c are not clearly dominated by the same influence of Pauli repulsion, as the sign of the electrostatic interaction was already repulsive in around half of the identified pi–pi contacts. It can also be seen that 4 of the systems retain an attractive contribution from their frozen energy, implying that the electrostatics were so attractive that they dominate even over Pauli repulsion at short range. These results suggest that the ion–ion interactions remain sufficiently nuanced such that it may not be sensible to lump them in with typical pi–pi interactions despite the marked similarity between the ternary diagrams when the Pauli term is included. Instead, both diagrams in Figure 6c and Figure 7c should be considered when classifying pi–pi interactions, and ion–ion interactions retain sufficient differences in Figure 6c to discount them as typical pi-stacking motifs.

A notably tunable example of ion–ion contacts is the Arg dimer motif, which has been identified as a frequent component of protein architecture.^8^ We find that every Arg dimer in our data set is highly repulsive in the gas phase but energetically bound in polar solvent. This is in alignment with previous results that suggest that Arg dimers become stable at a dielectric of 46.8 (DMSO), at which point the electrostatic environment supplied by PCM is effectively quenched.^68^ This has strong implications regarding the most likely places to find Arg–Arg contacts in proteins, namely, that they require an additional residue or exposure to solvent that aids in stabilizing the interaction. Whereas neutral aromatic contacts are not influenced by the polarity of their surroundings and may thus be found anywhere in a protein with nearly equal probability, like-charged ion–ion contacts represent a distinct class of “polarity-assisted” pi–pi contacts in proteins that can be found only if their immediate environment facilitates the interaction. It is notable that neutral HS pi–pi contacts would fall under this polarity-assisted definition if the HS paradigm were physically relevant, as the quadrupole–quadrupole repulsion should be screened similarly to the cation–cation interactions of Arg–Arg, but due to the ambivalence of electrostatics in neutral pi–pi contacts, the CFH model seems to be a far more apt description.

### Pi–Pi Contacts Involving the Protein Backbone

**Figure 8:**
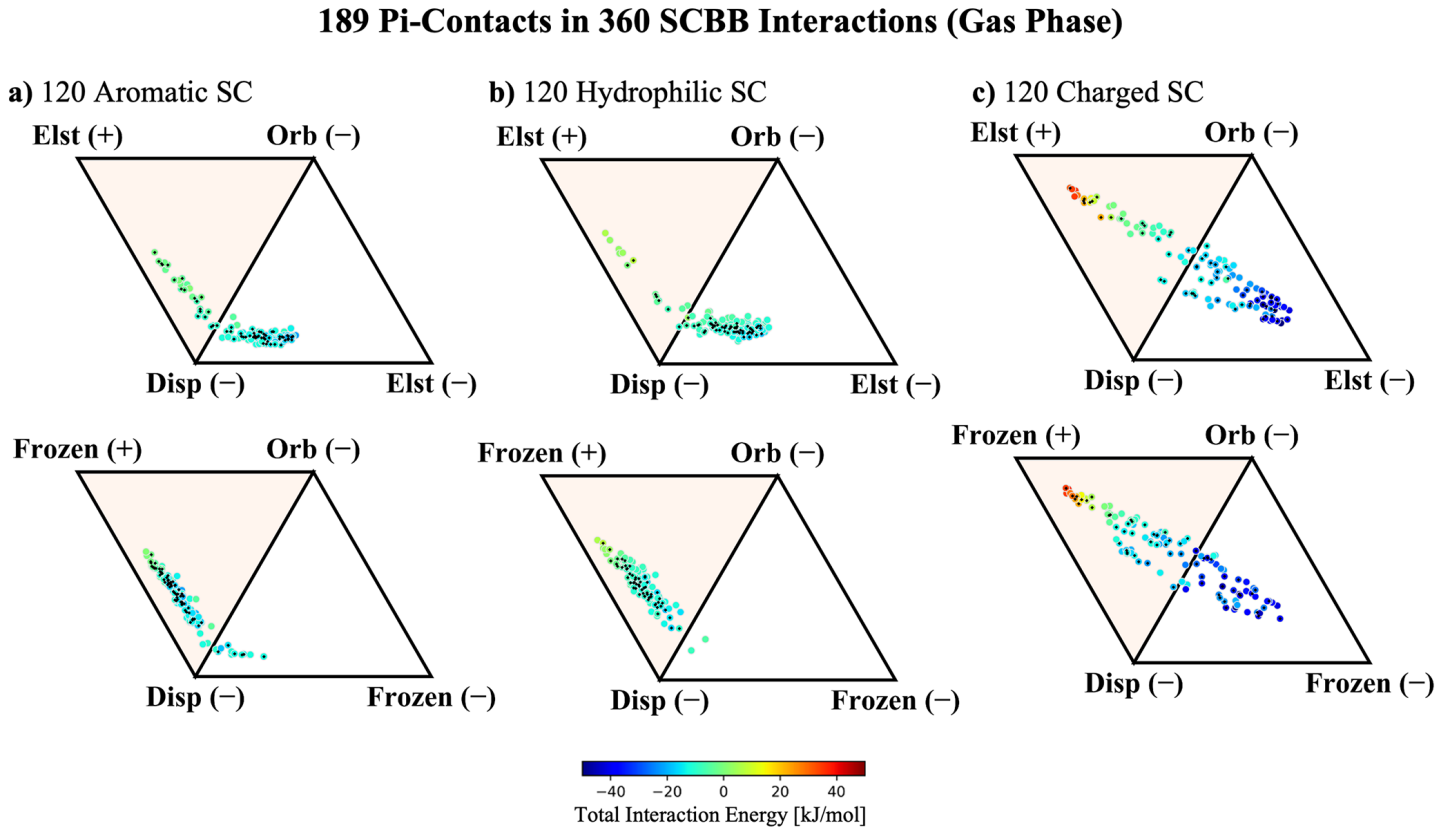
ALMO-EDA components for 360 SCBB fragments in the gas phase broken into 120 interactions with a) aromatic SC where total interaction energy ranges from −21 to 1 kJ/mol, b) hydrophilic SC where the total interaction energy ranges from −19 to 8 kJ/mol, and c) charged SC where the total interaction energy ranges from −52 to 35 kJ/mol. The 189 interactions with pi–pi contacts are marked with a black diamond; these include 67, 59, and 63 for aromatic, hydrophobic, and charged SC, respectively. The top row of diagrams considers only electrostatics in the signed vertices, while the bottom row adds Δ*E*_Pauli_ back into Δ*E*_Frz_.

With the most diverse subset of interactions fully characterized, we now consider the side-chain–backbone (SCBB) interactions in the gas phase in Figure 8. This set includes all of the variety of aromatic, hydrophilic, and charged moieties in the side chain subset interacting with the formamide group in methylacetamide fragments (the pi system present in the backbone subset). We break the SCBB set into subsets based on the type of functional group that contains the pi system in the SC fragment, leading to aromatic, hydrophilic, and charged distinctions in Figure 8a, b, and c, respectively. We continue to consider the two cases of *ΔE*_frz_ with and without Pauli repulsion.

For the aromatic subset, we again see a significant clustering of pi–pi contacts in the dispersion-dominated quadrant of the ternary diagram, with most pi–pi contacts exhibiting attractive electrostatics. A key difference in this data set is that there are more systems featuring repulsive electrostatics, i.e., there are more HS-type pi–pi contacts, but they are still in the minority by about a factor of 2. This may be unsurprising, as the C=O dipole moment of the methylacetamide fragments introduces a significant angular dependence on the interaction, where C=O systems whose bond dipole points into the aromatic ring on the SC fragment induce a straightforward dipole–quadrupole repulsion. The methylacetamide pi system also offers very little surface area to form favorable electron density overlap with the interacting SC fragment; therefore, charge penetration is less effective at counteracting the classically repulsive dipole–quadrupole repulsion. According to the bottom panel of Figure 8a, the inclusion of Pauli repulsion dramatically shifts the interaction profile in the aromatic SC subset. This is consistent with the behavior of neutral aromatic–aromatic systems that were analyzed in the SCSC systems, implying that the pi–pi interactions between methylacetamide and aromatic side chain systems are once again primarily van der Waals contacts and thus are remarkably consistent with typical pi–pi contacts.

Interestingly, the hydrophilic/methylacetamide interactions in Figure 8b are more straightforward. Like the hydrophilic–hydrophilic SCSC case, the hydrophilic SCBB subset shows a shift toward more favorable electrostatic interactions relative to that of the aromatic subset. This is likely to do with more favorable charge penetration interactions that emerge from breaking the 2D geometry of the SC pi system. When Δ*E*_Pauli_ is added back into the frozen energy contribution (Figure 8b, bottom), we again see a dramatic shift in the balance of the interaction profile toward a clean cut van der Waals picture that is dominated by dispersion and Pauli repulsion effects. Combining these results with those of the aromatic SC subset suggests that the van der Waals picture of pi-stacking once again leads to a consistent description of all neutral subsets of SCBB interactions.

The charged SC interactions in Figure 8c are immediately classifiable as something that resembles ion–pi interactions, with dispersion and orbital interactions dominating the interaction profile. The methylacetamide moieties supply slightly less favorable dispersion interactions than the pi systems found in the SCSC subset due to a smaller molecular surface area, leading to fewer closely interacting atoms. Thus, the *R*^–6^ dependence of the dispersion causes the balance to shift toward orbital interactions. The trend that these ion–pi interactions should remain relatively unperturbed by the inclusion of Pauli repulsion (as discussed for SCSC systems) is preserved in the SCBB subset, as shown in the bottom panel of Figure 8c. Overall, we find that the SCBB interactions are qualitatively similar to those in the SCSC subset, with the caveat that the smaller surface area of the methylacetamide pi system weakens those interactions that usually stabilize pi contacts, such as charge penetration and dispersion.

**Figure 9:**
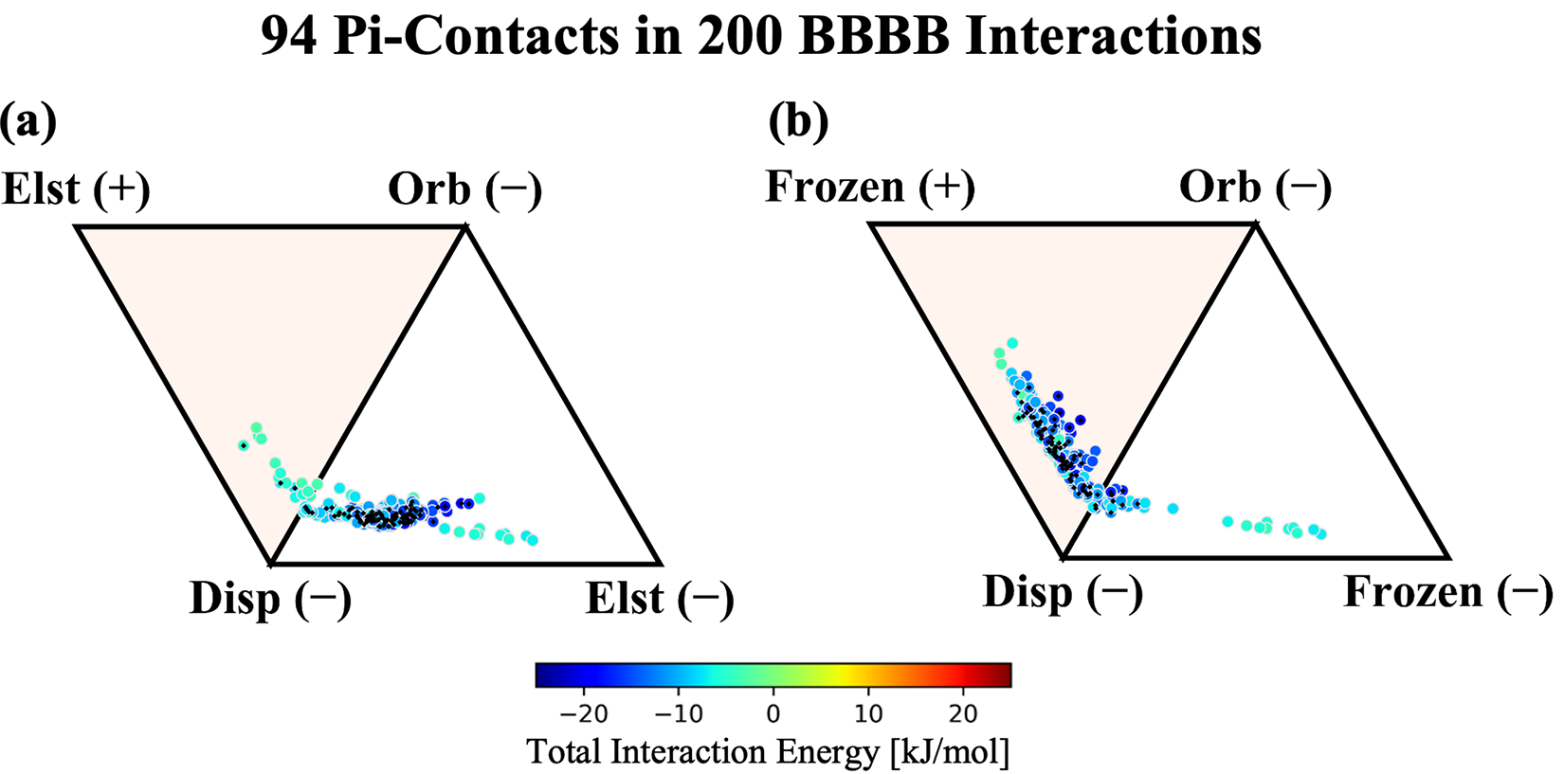
ALMO-EDA components for 200 BBBB fragments in the gas phase. For the BBBB gas phase, the total interaction energy ranges from −21 to −2 kJ/mol. Interaction profiles in the gas phase consider (a) only electrostatics and (b) electrostatics + Pauli repulsion in the signed vertices.

This is also evident when we consider the backbone–backbone (BBBB) subset of interactions, which comprise exclusively methylacetamide fragments. Perhaps surprisingly, the ALMO-EDA energy components for the BBBB subset displayed in Figure 9a show a significant clustering of pi–pi contact geometries that are largely dispersion-dominated with attractive electrostatics. Aside from the substantial dispersion contribution, the electrostatics can be explained from two vantage points. Classically, the formamide moieties in the BBBB fragments are more likely to interact in a configuration with antialigned dipole moments,^69^ thus promoting electrostatic attraction even in the multipole picture. Additionally, the quantum effect of charge penetration likely still contributes to the stability in the systems that meet the VGC distance threshold to qualify as pi–pi contacts. If we extend the HS picture to be more loosely defined as a “multipole model” of electrostatics rather than considering only the quadrupole moment, then both the HS and the CFH models will arrive at the same conclusion in BBBB systems. However, the importance of steric effects is considered only in the latter of the two models.

Although the electrostatic picture remains ambiguous, reincorporating the Pauli repulsion term into the frozen energy, as done in Figure 9b, sheds light on the contributions from the van der Waals interactions. We find that the methylacetamide dimers appear to interact within the parameters of the van der Waals model, exhibiting a uniform shift from attractive electrostatics to a repulsive frozen energy, despite their favorable antialigned dipoles. This is remarkably consistent with the results for neutral aromatic–aromatic interactions in the SCSC set, and implies that Pauli repulsion and dispersion dominate in BBBB interactions. The strong dependence of the interaction profile on Pauli repulsion implies that there is indeed substantial overlap of charge densities in the BBBB systems, and thus that charge penetration should dominate the electrostatic interactions as well, even though the sign of the electrostatics might be justified classically. Overall, the trend in BBBB interaction profiles appears to agree with the interactions found in purely pi-stacking systems, suggesting that close-contact methylacetamide groups are likely an overlooked candidate for pi-stacking interactions within the low dielectric interiors of proteins.

**Figure 10:**
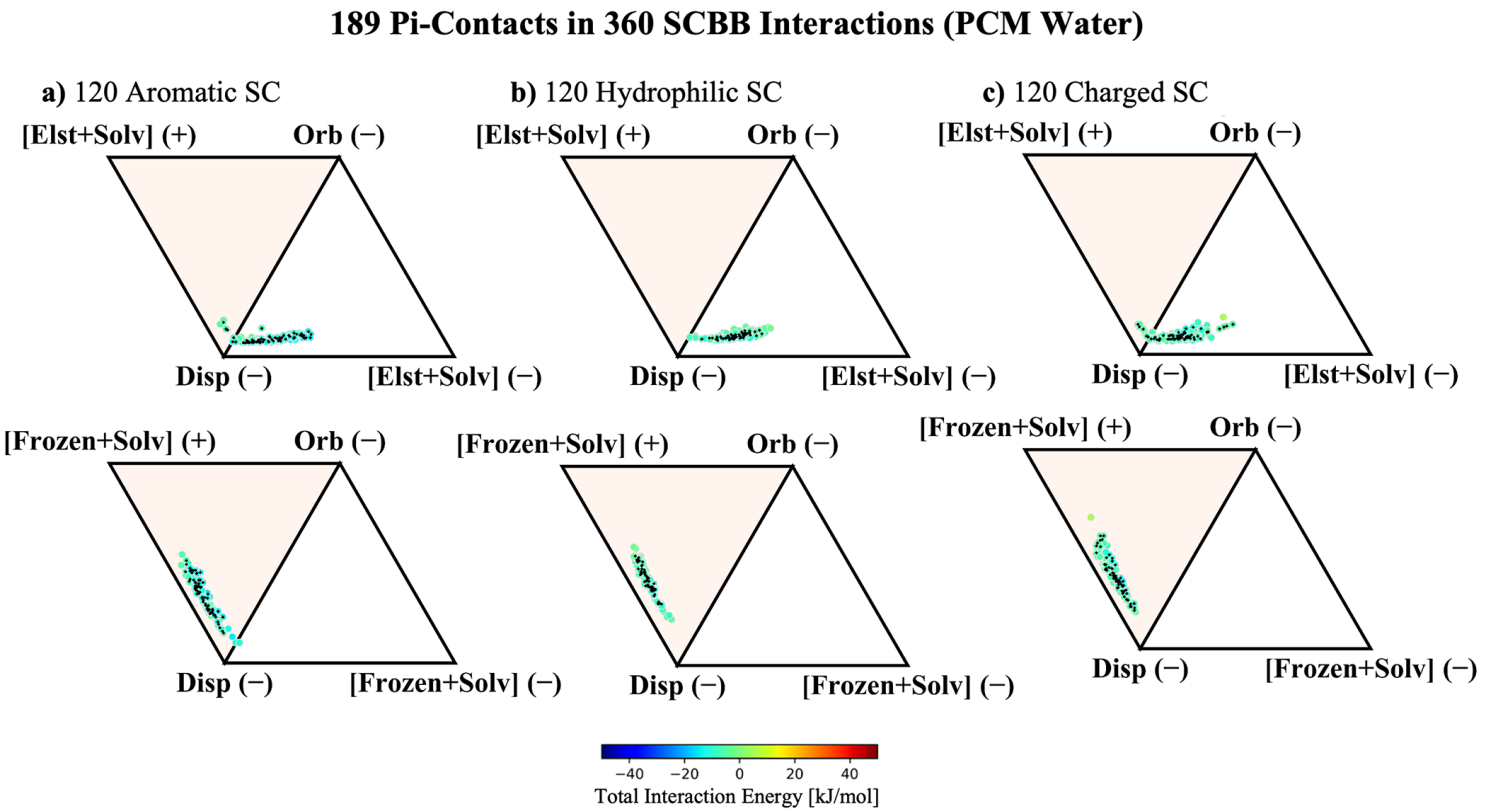
ALMO-EDA components for SCBB fragments in PCM water. Broken into 120 interactions with a) aromatic SC where total interaction energy ranges from −15 to −3 kJ/mol, b) hydrophilic SC where the total interaction energy ranges from −11 to −1 kJ/mol, and c) charged SC where the total interaction energy ranges from −13 to 5 kJ/mol. The 189 interactions with pi–pi contacts are marked with a black diamond; these include 67, 59, and 63 for aromatic, hydrophobic, and charged SC, respectively. The top row of diagrams considers only electrostatics in the signed vertices, while the bottom row adds Δ*E*_Pauli_ back into Δ*E*_Frz_. This is the PCM counterpart of Figure 8. Similar plots with a dielectric constant of 2.0 are provided in Figure S5.

**Figure 11:**
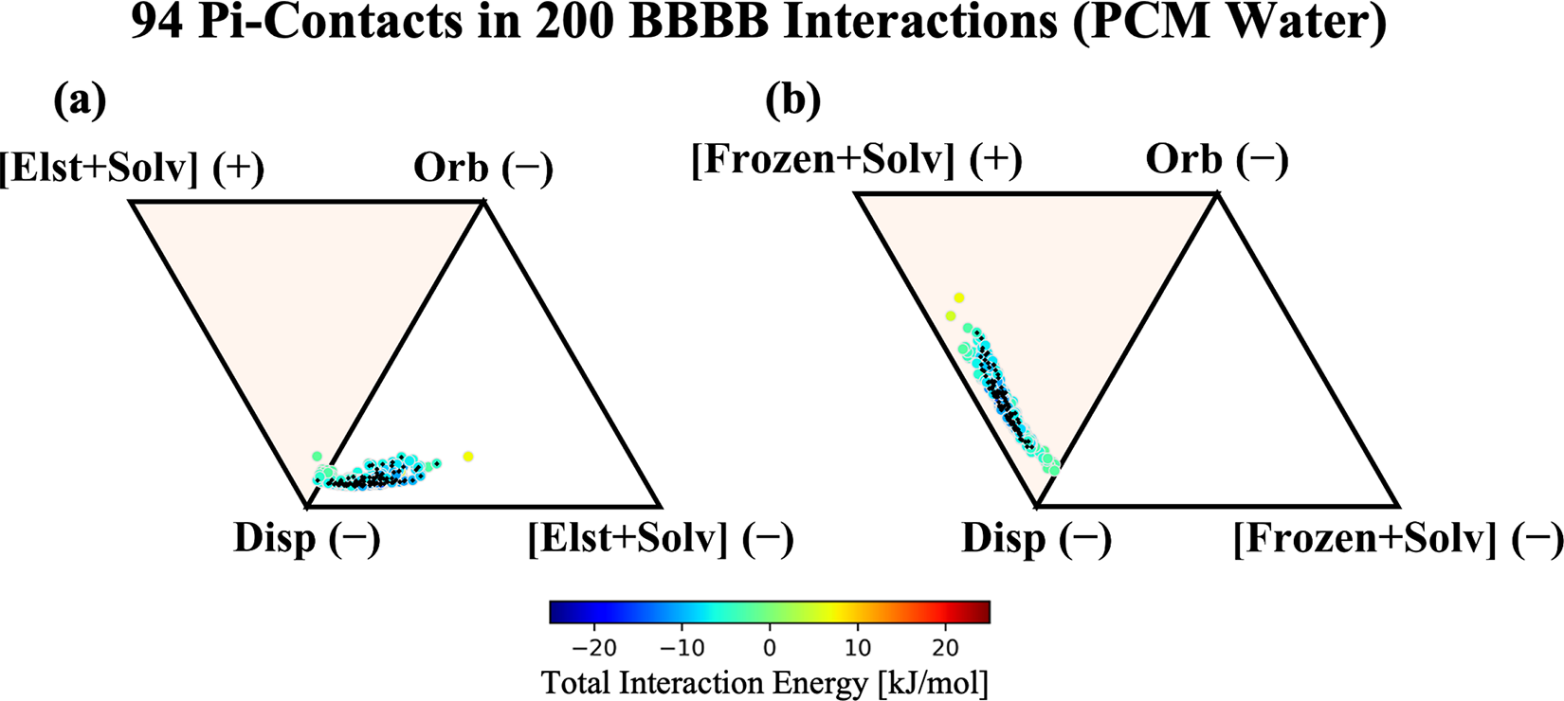
ALMO-EDA components for BBBB fragments in PCM water. Total interaction energy ranges from −15 to 7 kJ/mol. Interaction profiles consider (a) only electrostatics + solvent and (b) electrostatics + Pauli repulsion + solvent in the signed vertices. The 94 interactions identified as pi–pi contacts are marked with a black diamond. This is the PCM water counterpart of Figure 9. Similar plots with a dielectric constant of 2.0 are provided in Figure S6.

The SCBB and BBBB analyses within a dielectric environment can be considered simultaneously as their trends follow from the analysis of the SCSC subset. The results in Figure 10, which only feature neutral–neutral and ion–pi SCBB interactions, all engage in nominal CFH-type pi-stacking interactions within a high solvent dielectric. A key difference in the SCBB data set is that there are more systems featuring repulsive electrostatics; i.e., there are more HS-type pi–pi contacts, but they are still in the minority by about a factor of 2. These same trends can also be seen clearly in Figure 11 for BBBB interactions. Because CFH-type pi–pi contacts can be expected to be relatively immune to changes in solvent dielectric while HS-type contacts should change quite dramatically, inclusion of solvent dielectric actually clarifies the nature of the BBBB gas-phase results, which did not yield a straightforward interpretation when only considering electrostatics. Namely, the quenching of electrostatic interactions in solvent (particularly multipolar electrostatics) combined with the invariance of the BBBB interactions to PCM reveals that the BBBB interactions are more consistent with the van der Waals model as is evident in Figure 11a and 11b.

## Conclusions

Through a combination of molecular dynamics simulations and high-level quantum chemistry calculations, we have studied the relationship between Vernon’s geometric definition of pi–pi contacts in proteins and the fundamental molecular physics underpinning pi-stacking interactions. Using ALMO-EDA to decompose the interaction energies of the identified pi–pi contacts, we found unambiguously that attractive electrostatics are pervasive in Phe, Tyr, Trp, and His interactions among themselves and with amide groups in Asn, Gln, and methylacetamide representing the backbone. This finding, while consistent with previous studies of Phe–Phe interactions, is antithetical to the Hunter–Sanders model of pi–pi interactions, which hinges on repulsive quadrupole electrostatics. Instead, we find that when Pauli repulsion is considered, the interaction profile between neutral pi–pi contacts is guided predominantly by dispersion and Pauli repulsion (the van der Waals interactions). This is a key result, as the paradigm in biochemistry textbooks is rooted in the Hunter–Sanders definition, but we find that only roughly 3% of all pi–pi contacts are consistent with this model while the vast majority are consistent with the van der Waals model of Carter-Fenk and Herbert.^32,33^ Additionally, our discoveries reveal that interactions involving a charged residue with a neutral one can be characterized as ion–pi interactions. In nonpolar environments, we find ion–pi interactions to be distinct from pi-stacking because the dominant interaction components (electrostatics, orbital interactions, and dispersion) do not change when Pauli repulsion is considered. However, in polar environments, these ion–pi interactions lose their contributions from orbital interactions due to electrostatic screening, giving way to typical van-der-Waals-type pi–pi interactions, akin to those of neutral systems.

These findings may be used to inform additional physical parameters that could guide next-generation force field design for higher-fidelity modeling of pi–pi interactions in proteins. Assuming the QM energy and forces can be “decomposed” based on sound chemical principles such as EDA,^39^ a position long formulated within classical force fields which are also piecewise decomposable by design,^70,71^ nonbonded interactions can be better described. Advanced force fields are introducing new functional forms for charge penetration, charge transfer, and anisotropic polarization,^72,73^ and using QM cluster data (such as the protein fragment cluster data provided here), to maintain strict adherence to the many-body expansion.^74^ Because pi–pi contacts are defined by a balance among nonbonded interactions with greater subtlety depending on type and environmental considerations, they should provide an ideal stress test for advanced force field development.

The sheer abundance of CFH-type pi–pi contacts that we have found implies that pi–pi contacts should be ubiquitous in proteins, and the persistence of these interactions despite a myriad of protein and solvent environments could justify their important role in the formation of protein condensates.^8^ We have shown that interactions between two charged residues behave more like simple ion–ion interactions than pi–pi contacts in nonpolar media. However, in polar environments, we see the stabilization of like-charged pi–pi contacts such as the Arg dimer, which exhibits a CFH-type pi–pi interaction profile that is dominated by dispersion and Pauli repulsion under these conditions. In this regard, Lin et al. have shown when all the tyrosine (Tyr) residues of protein FUS (fused in sarcoma) low-complexity region where replaced with leucine (Leu), the phase separation was inhibited.^75^ In addition, Brady et al. have shown that substitution of all arginine (Arg) residues with lysine (Lys) in the N-terminal low complexity region of Ddx4 blocks phase separation.^76^ These pi–pi interactions, including “polarity-assisted” contacts, appear to be fundamental to the architecture of not only single proteins but may also shed light on an essential role in the formation and stability of biomolecular condensates.

## Data Availability

All the dimer geometries and ALMO-EDA data used in this manuscript are shared as PDB and JSON files at https://github.com/THGLab/PiContact

## Supporting Information Available

The number of dimers evaluated, parity plots, change in the number pi-contact when changing VGC parameters, as well as additional ternary diagrams for analyzing interactions in a PCM environment with a dielectric constant of 2 (PDF)
