# Supplementary material for: The Energetic Origins of Pi–Pi Contacts in Proteins
Source: J Am Chem Soc. 2023 Nov 2;145(45):24836–51. doi: 10.1021/jacs.3c09198 (PMC10655088; doi:10.1021/jacs.3c09198)
Supplement: Supplementary file 1 — ja3c09198_si_001.pdf [file ja3c09198_si_001.pdf]

Supplementary Information: The Energetic Origins of Pi-Pi Contacts in Proteins

Kevin Carter-Fenk[1,2,§], Meili Liu[1,2,5,§], Leila Pujal[6], Matthias Loipersberger[1,2], Maria Tsanai[1,2], Robert M. Vernon[7,8], Julie D. Forman-Kay[7,8], Martin Head-Gordon[1,2], Farnaz Heidar-Zadeh[6,9,§], Teresa Head-Gordon[1−4*]

[1]*Kenneth S. Pitzer Center for Theoretical Chemistry,* [2]*Department of Chemistry,*
[3]*Department of Chemical and Biomolecular Engineering,* [4]*Department of Bioengineering,*
University of California, Berkeley, California 94720, USA
[5]*Department of Chemistry, Beijing Normal University, Beijing 100875, China,*
[6]*Department of Chemistry, Queen's University, Kingston, Ontario K7L 3N6, Canada*
[7]*Molecular Medicine Program, Hospital for Sick Children, Toronto, Ontario M5G 0A4, Canada*
[8]*Department of Biochemistry, University of Toronto, Toronto, Ontario M5S 1A8, Canada*
[9]*Center for Molecular Modeling, Ghent University, B-9052 Ghent, Belgium*
§*These authors contributed equally*

E-mail: farnaz.heidarzadeh@queensu.ca,mhg@cchem.berkeley.edu,thg@berkeley.edu

Table S1: Number of side-chain side-chain (SCSC), side-chain backbone (SCBB), and backbone-backbone (BBBB) interactions sampled from each protein. The number in parentheses specifies how many of those interactions have a pi-contact.

Protein	BBBB	SCBB	SCSC
**1vex**	80 (30)	170 (83)	80 (27)
**2xr6**	50 (12)	100 (52)	280 (122)
**1arb**	70 (52)	90 (54)	250 (107)
**Total**	200 (94)	360 (189)	610 (256)

Table S2: Effect of 3 pi-contact parameters (i.e., the first 3 columns) on the number of pi-contacts (i.e., last column) identified among 280 SCSC interactions of 2XR6. The first row of the table corresponds to the original VGC definition of pi-contact.

cos(angle)	distance	icut	count
0.8	1.5	2	122
0.8	**1.6**	2	129
0.8	**2.0**	2	150
0.8	**2.5**	2	161
0.8	1.5	**1**	155
**0.75**	1.5	2	133

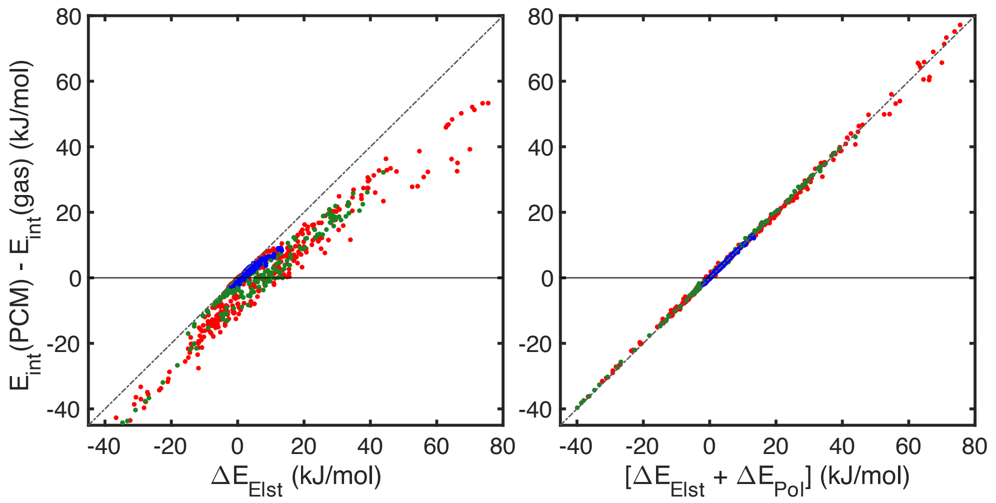

Figure S1: Parity plots for the difference in total interaction energy (y-axis) and (left) the difference in electrostatic energy or (right) the difference in electrostatic and polarization energies between their respective values in gas-phase and PCM ($\varepsilon = 78.39$). The differences in energy components were computed as follows: $\Delta E_{\text{int}} = E_{\text{int}}(\text{PCM}) - E_{\text{int}}(\text{gas})$, $\Delta E_{\text{Elst}} = E_{\text{Elst}}(\text{PCM}) - E_{\text{Elst}}(\text{gas})$, and $\Delta E_{\text{Pol}} = E_{\text{Pol}}(\text{PCM}) - E_{\text{Pol}}(\text{gas})$. The solvation energy ΔE_{solv} was incorporated into $E_{\text{Elst}}(\text{PCM})$. The data are color coded by (red) SCSC, (green) SCBB, and (blue) BBBB subset.

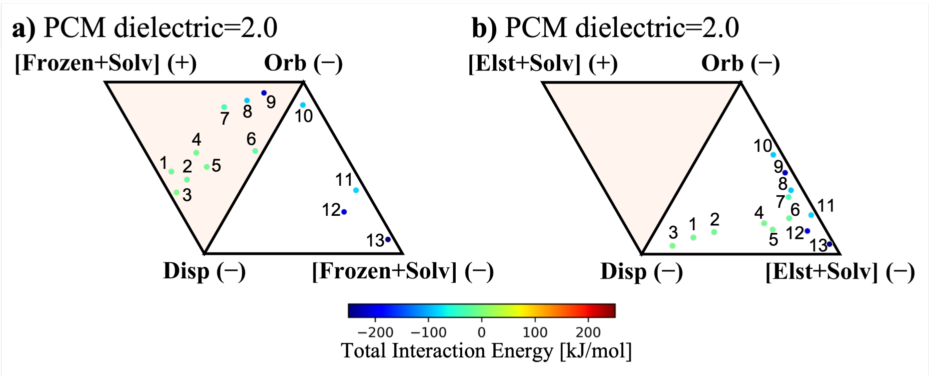

Figure S2: *Ternary diagrams corresponding to Figure 2 with dielectric=2.0 environment from PCM.* Ternary diagrams utilizing the (a) Frozen (Frz) interaction and (b) permanent electrostatics (Elst) interaction.

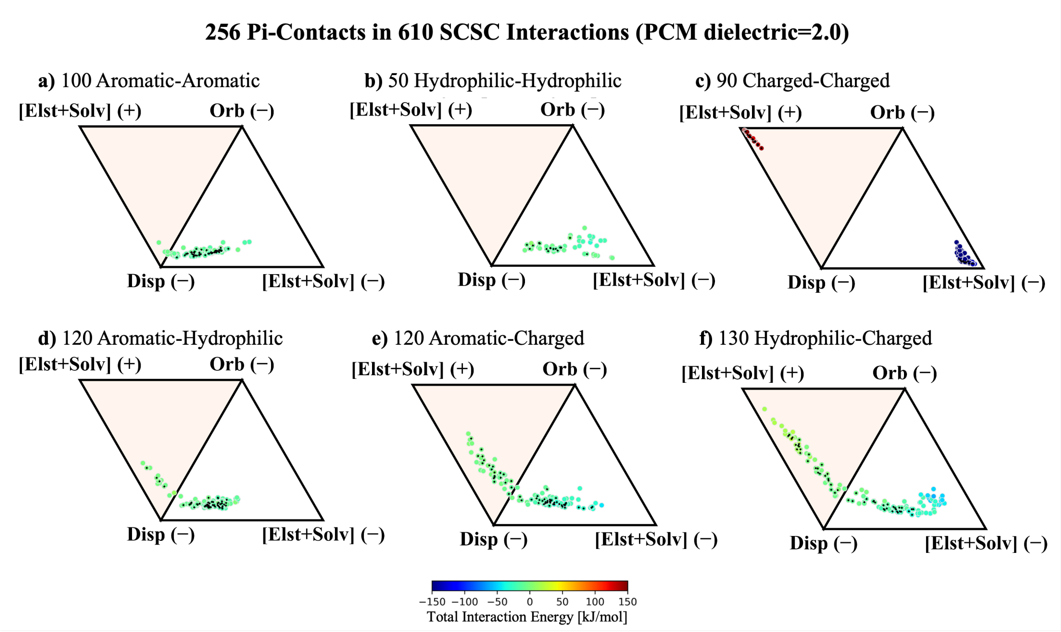

Figure S3: *Ternary diagram corresponding to Figure 6 for SCSC interactions with dielectric=2.0 environment from PCM broken into dispersion, orbital and electrostatic interactions.* The 256 interactions with pi-pi contacts are marked with a black diamond.

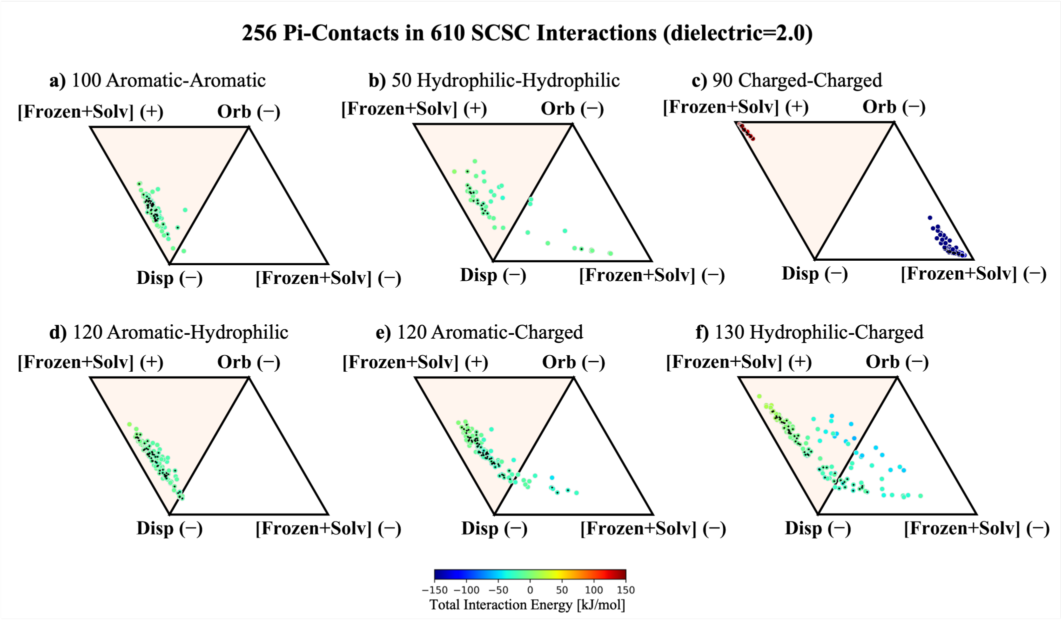

Figure S4: *Ternary diagram corresponding to Figure 7 for SCSC interactions with dielectric=2.0 environment from PCM broken into dispersion, orbital, and frozen interactions.* The 256 interactions with pi-pi contacts are marked with a black diamond.

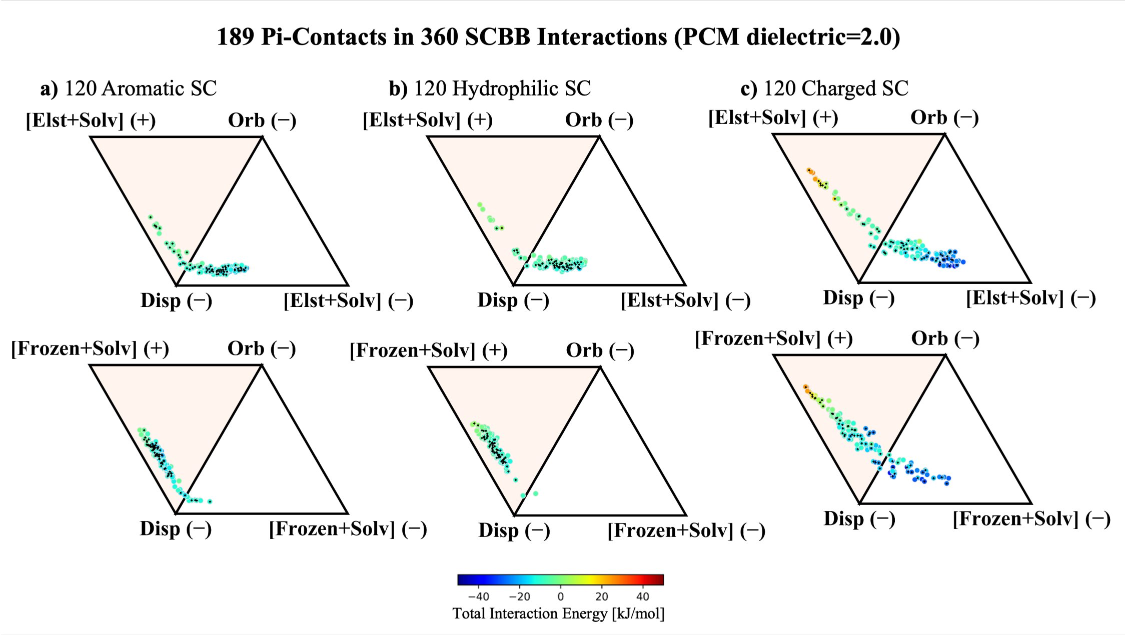

Figure S5: *Ternary diagram corresponding to Figure 10 for SCBB interactions with dielectric=2.0 environment from PCM.* Broken into 120 interactions with a) aromatic SC , b) hydrophilic SC , and c) charged SC The 189 interactions with pi-pi contacts are marked with a black diamond; these include 67, 59, and 63 for aromatic, hydrophobic, and charged SC, respectively. The top row of diagrams considers only electrostatics in the signed vertices, while the bottom row adds E_{Pauli} back into E_{Frz}.

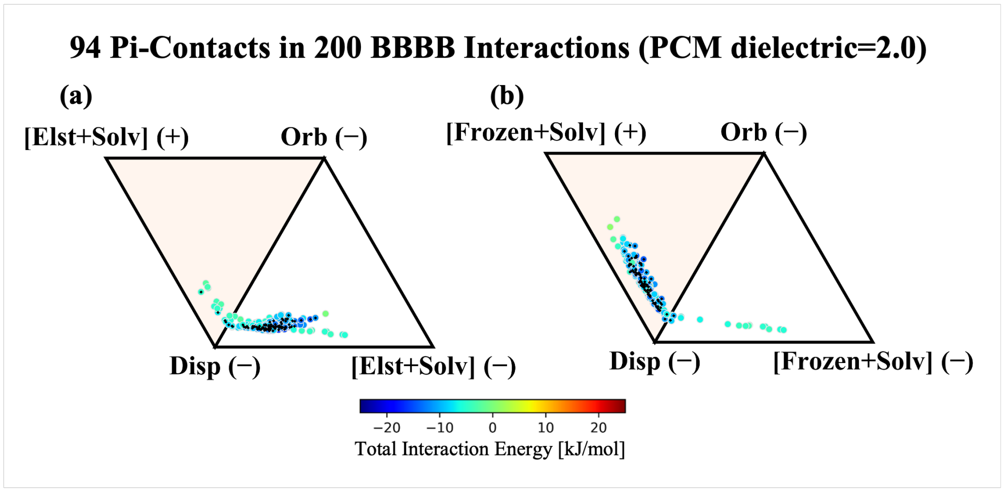

Figure S6: *Ternary diagram corresponding to Figure 11 for BBB interactions with dielectric=2.0 environment from PCM.* Ternary diagrams utilizing the (a) Frozen (Frz) interaction and (b) permanent electrostatics (Elst) interaction.
